# Food environment and childhood obesity: the effect of dollar stores

**DOI:** 10.1186/s13561-015-0074-2

**Published:** 2015-12-02

**Authors:** Andreas C. Drichoutis, Rodolfo M. Nayga, Heather L. Rouse, Michael R. Thomsen

**Affiliations:** 1 Department of Agricultural Economics & Rural Development, Agricultural University of Athens, Iera Odos 75, Athens, 11855 Greece; 2 Department of Agricultural Economics & Agribusiness, University of Arkansas Division of Agriculture, Fayetteville, 72701 AR USA; 3 Norwegian Institute of Bioeconomy Research, Ås, Akershus, Norway; 4 Korea University, Seoul, South Korea; 5 Department of Human Development and Family Studies, Iowa State University and Arkansas Center of Health Improvement, University of Arkansas for Medical Sciences, Little Rock, 72205 AR USA

**Keywords:** D10, I10, C31, C33, R10, Childhood obesity, Food-at-home, Propensity score matching, Difference-in-differences

## Abstract

In this paper we examine the effect of dollar stores on children’s Body Mass Index (BMI). We use a dataset compiled by the Arkansas Center for Health Improvement that reflects a BMI screening program for public school children in the state of Arkansas. We combine propensity score matching with difference-in-differences methods to deal with time-invariant as well time-varying unobserved factors. We find no evidence that the presence of dollar stores within a reasonably close proximity of the child’s residence increases BMI. In fact, we see an increase in BMI when dollar stores leave a child’s neighborhood. Given the proliferation of dollar stores in rural and low-income urban areas, the question of whether dollar stores are contributing to high rates of childhood obesity is policy relevant. However, our results provide some evidence that exposure to dollar stores is not a causal factor.

## Introduction

At present, nearly 35 percent of young Americans aged 6 to 19 are overweight and 19 percent are obese [1]. This is up from just over 4 percent in the 1960s [2]. In Arkansas, the problem is more pronounced. Twenty one percent of Arkansas schoolchildren are obese and many more are at risk of obesity [3]. In fact, only 60 percent of Arkansas schoolchildren have a healthy weight status. The childhood obesity problem has caught the attention of policy makers at all levels of government and has become a front-burner issue for concerned community and business leaders. Proposals to address childhood obesity are often aimed at augmenting features of the environment by improving access to healthy foods in or around the home and school, reducing accessibility and exposure to unhealthy food, and/or providing more opportunities for exercise and vigorous play. For example, many of the strategies proposed by the Institute of Medicine [4] to address obesity emphasize the built environment, the commercial food environment, and the food distribution system. Similarly, Frieden et al. [5] call for neighborhood policy interventions to encourage healthy food choices. Specifically, they advocate for changes that increase the likelihood that healthy foods will be chosen by default. Goldberg and Gunasti [6] provide recommendations aimed at the food marketing system both in terms of promotional messaging and in terms of product design, pricing, and distribution.

Ambitious and comprehensive interventions are clearly needed to reduce the incidence of childhood obesity. However, concerns have been expressed that existing research is inadequate to guide policy interventions. For example, Story et al. [7] acknowledge that the systematic study of interactions between features of the environment, policy interventions, and nutrition outcomes is a relatively new field of study. As such, it lacks well established models and faces numerous challenges in terms of measurement of environmental attributes and empirical design. Researchers attempting to investigate the link between environmental attributes and obesity face important challenges. First, the environmental features of interest are likely to be endogeneously determined with rates of obesity. For instance, food stores would be expected to consider consumer demand when making choices about the location of stores, but consumers with stronger demand for unhealthy foods may be making lifestyle choices that otherwise place them at a higher risk of obesity [8]. Neighborhood choice is also not randomly assigned [9]. Thus, if health-conscious individuals self-select into neighborhoods that are conducive to healthy diets or active lifestyles, the statistical association between neighborhood features and obesity is suspect. Second, the impact of environmental features may be context specific. For example, in one context a new food store may meaningfully expand healthy food options for residents and facilitate healthy dietary choices. In another, the increased competition that results from the additional store may have the opposite effect by lowering prices on less healthy foods [10]. For these reasons, it is not surprising that it has been difficult to draw clear conclusions from correlational studies on the relations between features of the environment and weight outcomes.

The aim of this article is to examine the role of dollar stores. Dollar stores are an unstudied feature of the built environment that may impact childhood obesity, especially in predominantly rural states such as Arkansas. In comparison to supermarkets, dollar stores provide a very narrow range of food items, but at price points much lower than convenience stores and often lower than supermarket prices. A recent inventory of Arkansas dollar stores found very limited offerings of healthier (e.g., lower-sodium) product formulations and limited offerings of fresh fruits and vegetables [11].

**Fig. 1:**
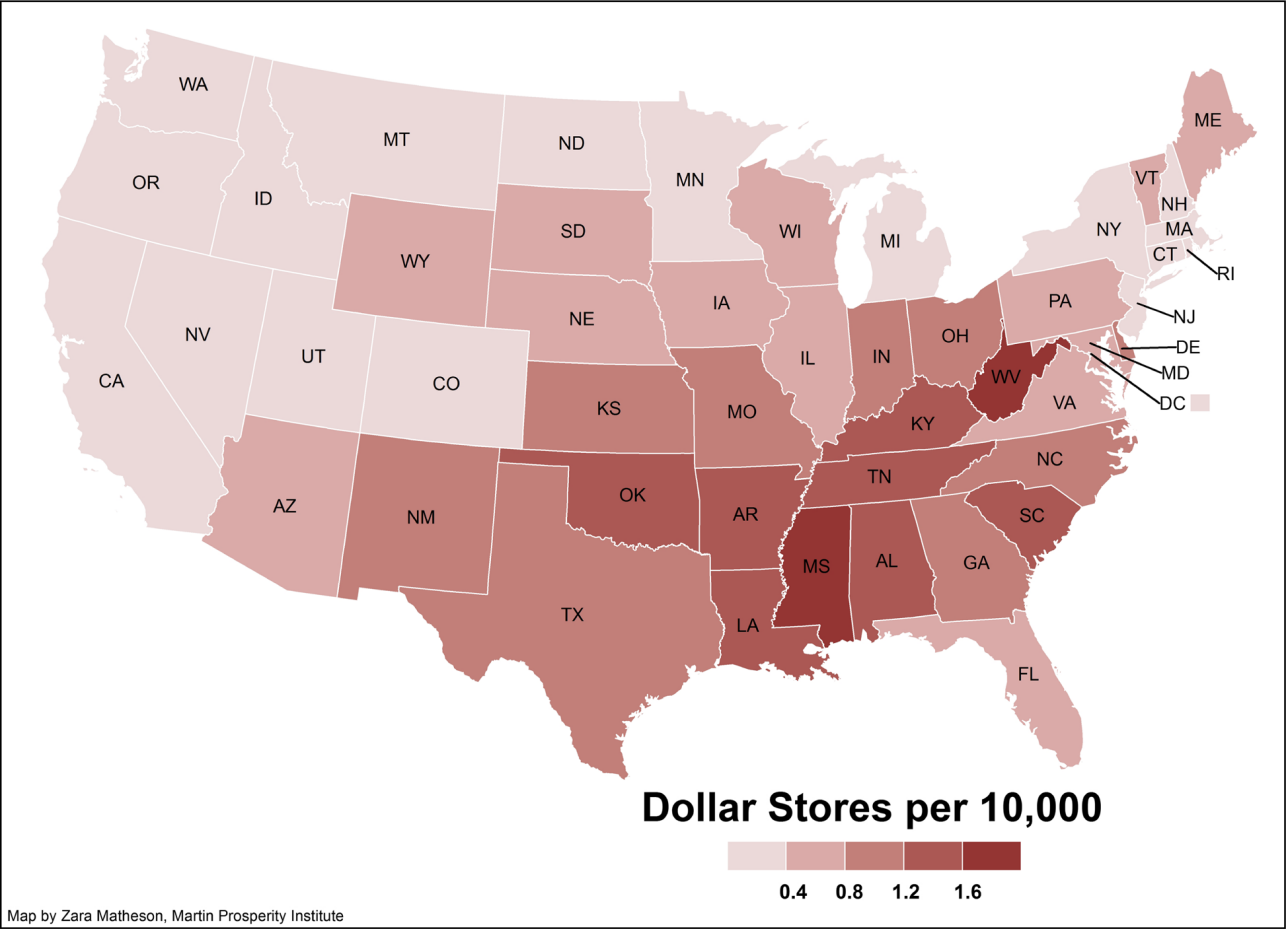
Dollar stores distribution map across US. Source: The Martin Prosperity Institute, Joseph L. Rotman School of Management, University of Toronto. Map created by Zara Matheson

Dollar stores have been growing markedly throughout the United States but this growth has not been uniform. Figure 1 [12] shows dollar stores distribution across US. The mid South is one region where dollar stores are becoming prominent features of the retail environment. Natunewicz [13] provides counts, by state, for the four leading dollar store retailers. A simple adjustment of these data by population reveals that Arkansas, Mississippi, and Louisiana each have more than 140 dollar stores per million residents. This compares to only 14 stores per million residents in California and 37 stores per million residents in New York State. Even in Texas, dollar store density is considerably smaller at 86 stores per million residents. Dollar stores are not only a rural phenomenon. These stores are also growing in urban areas, albeit in less desirable neighborhoods [13].

As a result, a larger fraction of the household budget has been shifted toward dollar stores and the trend is not confined to less affluent households. Even among households with an income of at least $75,000, 28 % now spend more in the dollar channel [14]. According to one industry report, the dollar (and variety) store industry capitalized on the recession to attract more middle class consumers making it a $62bn business that has seen a 3.5 % annual growth in the period 2009–2014 [15]. The dollar store channel has seen the largest year-over-year share increase in shopping visits (as compared to the brick-and-mortar market overall), likely driven in part by new store openings, where in one recent retail quarter (May 2014 – July 2014) shopping visits were up 14 % with a particular increase in the 16–24 age group [16].

Given the significant increase in the number of dollar stores, our objective in this study is to examine how access to these types of stores influences weight outcomes of children. Our empirical strategy involves a difference in differences (DiD) framework coupled with propensity score matching. The National Research Council [17] has called for strong quasi-experiments that couple observational data with one or more empirical identification strategies to improve understanding of the factors that may be responsible for the growth in obesity rates. Our focus is on childhood obesity outcomes among early elementary schoolchildren in Arkansas. Arkansas provides an ideal context within which to conduct this research. As already noted, it has one of the highest childhood obesity rates in the country. However, the state has been taking active steps to address this problem and has assembled unique panel datasets of childhood Body Mass Index (BMI) screenings that can be used to assess the impact of environmental features such as dollar stores.

## Background

Arkansas was the first state to require BMI measurements for all public schoolchildren. The Arkansas General Assembly passed Act 1220 of 2003, which established a formal Child Health Advisory Committee (CHAC) and mandated BMI screenings for public schoolchildren. Our data on weight outcomes are from the Arkansas BMI dataset for 2004 through 2010. These data are maintained through legislative mandate at the Arkansas Center for Health Improvement (ACHI) [18]. The data contain age-gender specific z-scores and are based on height and weight measurements taken by trained personnel within the public schools. Weight and height of school children were measured yearly in all grades beginning with the 2003–2004 school year but in 2007 this was changed to measurement only of children in even grades. Hence, the dataset we use is an unbalanced panel which contains information for schoolchildren from 2004 to 2010.

Dollar store location data were obtained from Dun and Bradstreet (D&B) for the period 2004 through 2010. To ensure that BMI screenings in any given year were matched correctly to the locations of dollar stores as they existed in that year, we obtained archival data showing the location of dollar stores as of December of the year in question. ACHI personnel geocoded student addresses within the BMI dataset and linked them geographically to the D&B data on dollar store locations. The final dataset contains measures of the food environment around the children’s home and schools such as number of fast food restaurants, dollar stores, convenience stores and grocery stores within a certain radius of the child’s home.

ACHI personnel also matched the BMI screenings to neighborhood demographic characteristics from the 2009 American Community Survey (ACS) block-group summary file. The 2009 ACS reflects an average over the 2005–2009 period and so is centered on the 2004 to 2010 period covered by the BMI data we use here. The ACS data provide information on socioeconomic characteristics of the census block group where the student lives as well as information on neighborhood characteristics such as the proportion of population by race, income level, education, and work status.

## Methods

In this study we examine the effect of access to dollar stores (DS) on children’s BMI. To determine whether children and their guardians have easy access to dollar stores, we created binary measures of whether a dollar store is in close proximity to the child’s residence. For this reason we adopted one of the measures that the Economic Research Service (ERS) of the US Department of Agriculture (USDA) uses to define food desert areas i.e., distance to the nearest store, taking into account that the definition applies differently to urban and rural areas.^1^ Therefore, a child was considered exposed to a DS (i.e., has easy access to the store) if there was at least one store within a one-mile radius from the child’s residence in an urban area or one store within a ten-mile radius of the child’s residence in a rural area. Otherwise, the child was considered non-exposed (i.e., did not have easy access to a DS).

For reasons that will become apparent momentarily, we only use cohorts of students that we observe for a full five-year period. Given that the dataset we use extends through 2010, this implies that our sample includes three different age cohorts i.e., 2004 to 2008, 2005 to 2009 and 2006 to 2010.^2^ We also limit our analysis to school children who were kindergarten in their first year of their respective age cohort. Thus, by construction, the kindergarten cohort is observed up to the 4th grade. We focus specifically on children in early elementary grades because their diets are more likely to be dictated by the adults in their lives and so any DS effects would most likely be felt in these young children. For children at later elementary grades, a number of other confounding factors could potentially be contributing to their weight. Nevertheless, this could also be an interesting topic for future investigation. The cohorts used in the analysis are depicted in Table 1.

**Table 1:** Cohorts used in the study

	Years						
	2004	2005	2006	2007	2008	2009	2010
2004 cohort	✓	✓	✔		✔		
	K	1^st^ grade	2^nd^ grade		4^th^ grade		
2005 cohort		✓	✓	✔		✔	
		K	1^st^ grade	2^nd^ grade		4^th^ grade	
2006 cohort			✓	✓	✔		✔
			K	1^st^ grade	2^nd^ grade		4^th^ grade

To examine the effect of ease of access to a DS, we use the panel difference-in-differences (DiD) method. DiD estimation is a common approach in program and policy evaluations [19] and it has become a common strategy to estimate effects of programs that could impact nutrition, weight, or health outcomes [20, 21]. Given the four year subsamples with the cohorts exhibited in Table 1, we are able to examine two-year exposure to DS (i.e., ease of access) or two-year non-exposure to DS. Thus, we define the first two years of each age cohort as period 1 and the last two years of each cohort as period 2. We use two years for each period so that there is adequate time for any effect of the food environment to manifest itself. Table 1 marks period 1 with the ‘✓’ symbol and period 2 with the ‘✔’ symbol. The table also exhibits the grade level at which we observe each age cohort during each year. We then define two different treatments that we examine separately in the analysis. Our first treatment includes children that were exposed (i.e., had ease of access) to a DS in period 2 but were not exposed to a DS in period 1. Our control group in this case includes children that were not exposed to a DS in both periods. Our second treatment includes children that were not exposed to a DS in period 2 but were exposed to a DS in period 1. Our control group in this case includes children who were exposed to a DS in both periods. Treatments and control groups are depicted in Table 2 where we define by ‘E’ exposure (i.e., having at least one store within the radial distances described above) and by ‘N’ non-exposure. Treatment 1 will be referred to as the ‘Exposed’ treatment and treatment 2 as the ‘Non-exposed’ treatment.

**Table 2:** Treated and control groups by age cohorts

			Years						
			2004	2005	2006	2007	2008	2009	2010
2004 cohort	Treatment 1	Treated	N	N	E		E		
		Control	N	N	N		N		
	Treatment 2	Treated	E	E	N		N		
		Control	E	E	E		E		
2005 cohort	Treatment 1	Treated		N	N	E		E	
		Control		N	N	N		N	
	Treatment 2	Treated		E	E	N		N	
		Control		E	E	E		E	
2006 cohort	Treatment 1	Treated			N	N	E		E
		Control			N	N	N		N
	Treatment 2	Treated			E	E	N		N
		Control			E	E	E		E

Notes: *E* exposed, that is, there is at least one dollar store within a one mile radius (10 mile radius if child resides in a rural area), *N* non-exposed, that is, there is no dollar store within a one mile radius (10 mile radius if child resides in a rural area)

Exposure or non-exposure to a particular DS in period 2 may have been due to one of two rival explanations. If the child resides in the same location during period 1 and period 2, then exposure and non-exposure can be attributed to the fact that a DS opened or closed, respectively, within a radius distance from the child’s residence. On the other hand, if the child has moved to a different residence in period 2, then exposure (non-exposure) can be attributed to the child moving from an area without (with) a DS to an area with (without) this type of store. Thus, in addition to performing our analysis for the full sample, we repeat the analysis for two subsamples: (a) the ‘Movers’ which are defined as children that moved in a different residence in period 2 and (b) the ‘Stayers’ which are defined as children that did not move to a new residence in period 2.

Although the use of DiD is appealing due to its simplicity, the validity of a DiD estimate hinges upon the possible endogeneity of the intervention itself [22]. An additional assumption requires that in the absence of the treatment, the average outcomes for the treated and control groups would have followed parallel paths over time [23]. This latter assumption, known as common time effects (see for example Blundell et al. [24]), would be unattainable if, for example, pre-treatment characteristics associated with the dynamics of the outcome variable are unbalanced between the treated and control groups.

To alleviate concerns regarding the comparability of the treatment and control groups and to limit model dependence [25, 26], we use propensity score matching technique prior to running our panel DiD models. Heckman et al. [27] concluded that matching helps control for heterogeneity in initial conditions and also controls for unobserved determinants of participation. Blundell and Dias [28] also show that combining propensity score matching with DiD (MDiD) can be advantageous and has the potential to improve the quality of non-experimental evaluation results significantly. This is because DiD deals with time-invariant unobserved factors, while matching rebalances the sample to deal with time-varying unobserved factors [20]. Thus, the MDiD combines the advantages of both methods.

However, matching estimators hinge upon a significant assumption, the Conditional Independence Assumption (CIA), which requires that selection is on observables only. However, with MDiD there is scope for an unobserved determinant of participation as long as this can be represented by separable individual/time specific components in the error term. Blundell and Dias [28] show that CIA in MDiD can be replaced with a different assumption that only assumes that “…controls have evolved from a pre- to a post-programme period in the same way treatments would have done had they not been treated”. This occurs both in the observable component of the model and in an unobservable time trend. In addition, if the impact of the treatment is heterogeneous with respect to observable characteristics, we must guarantee that the distribution of the relevant observable characteristics is the same across periods and assignment to treatment for the evaluation to make sense. Blundell et al. [29] show how propensity score matching can ensure that the composition of the samples being compared is kept constant with respect to key determinants of outcomes before they apply a DiD estimator.

In our MDiD method, we first perform propensity score matching with the aim of balancing the distribution of observable characteristics between treated and control observations. We then apply DiD on the balanced sample. Matching is performed on the first year of BMI measurement of each cohort and propensity scores are estimated separately for each age cohort depicted in Table 1. The control variables for the PSM model include childrens’ gender, age (in months), race (Black/African-American, Hispanic/Latino or Native; White/Asian is the excluded category), language spoken at home (dummy if Spanish is spoken at home), an urban residence location dummy, dummies for free and reduced lunch participation (as proxies for income) as well as census-block group characteristics that capture neighborhood effects.^3^ Most importantly, the PSM model controls for relative distance of competing types of stores as well as number of competing stores within the given radius between neighborhood income and retail density.^4^ Although some of the variables above could be endogenous to the treatment, Lechner [30] showed that this would not matter as long as the usual formulation of the CIA holds.

Matching was performed with four different matching estimators that differ on how strict the matching process is: (1) two nearest neighbors without a caliper, (2) five nearest neighbors without a caliper, (3) two nearest neighbors with a caliper set at 1/4 of the standard deviation of the estimated propensity score, and (4) five nearest neighbors with a caliper set at 1/4 of the standard deviation of the estimated propensity score.^5^ After matching we estimate fixed and random effects DiD models using the matched samples. In terms of notation, the DiD estimate comes from a (random effects) model of the form:

(1) $$ \begin{aligned} BMI_{it}&=b_{0}+b_{1}Period_{it}+b_{2}Treat_{i}\\ &\quad+b_{3}Period_{it}\times Treat_{i}+\boldsymbol{{\gamma}X}_{it}+u_{i}+\varepsilon_{it}  \end{aligned}  $$

where *P* *e* *r* *i* *o* *d* is a dummy for the last two years where we observe each child (Period 2), *Treat* is a treatment dummy and ***X*** is a vector of controls as discussed above. The dependent variable is the Body Mass Index which has been calculated as a ratio (*w* *e* *i* *g* *h* *t*(*l* *b*)/(*h* *e* *i* *g* *h* *t*(*i* *n*))^2^)×703 and then converted to age-gender specific z-scores according to the Centers for Disease Control and Prevention guidelines [31]. Appropriate modifications to equation 1 are in place for the fixed effects counterpart.

## Results

### Balancing tests

Before examining the results, it is important to take a look at the performance of the matching estimators and the distribution of observable covariates (balancing) of the matched data. Table 5 in the Appendix shows results from balancing tests arranged in separate panels for ‘Movers & Stayers’, ‘Movers’ and ‘Stayers’. Results from all four matching estimators are reported in each panel. Although matching is performed for each age cohort separately, we report balancing tests after we pool together the matched observations from all age cohorts given that the DiD estimates come from the pooled age cohorts. Nothing changes, however, when we perform the balancing tests for each age cohort separately.

For each matching estimator and treatment (Exposed and Non-exposed) two *p*-values are reported in vertical orientation. The upper *p*-value corresponds to a likelihood-ratio (LR) test of the joint significance of all the regressors before matching. The lower *p*-value corresponds to a LR test of the joint significance of all the regressors after matching. A small *p*-value before matching (rows labeled as BM) indicates that the distribution of observables is not balanced between treated and control units, while a large *p*-value after matching (rows labeled as AM) indicates that balance has been achieved.

It is apparent across all panels of Table 5 in the Appendix that in all cases the distribution of covariates before matching was not balanced to begin with. After matching, balance has been achieved in most cases. There are only a couple of exceptions and these are marked with gray in Table 5 in the Appendix. The two exceptions concern exclusively the five nearest neighbor estimator. Therefore, caution is needed when interpreting results for this specific matching estimator.

Additional columns in Table 5 in the Appendix show mean standardized percent of absolute bias before and after matching.^6^ As depicted in the table, mean standardized percent absolute bias is generally higher before matching and lower after matching (even for the two cases of the five nearest neighbor estimator for which a good balance was not achieved). In general, a lower mean percent absolute bias is a sign that matching was able to reduce differences of observables between treated and control units.

Two additional columns in Table 5 in the Appendix show the number of treated and control observations before matching as well as the number of treated and control observations that are left after matching. To get a closer look at how exactly the matching worked in each case, Table 6 in the Appendix shows the number of observations that were dropped and retained after matching per cohort, treatment and matching estimator.^7^ More detailed information for the unmatched samples are provided in Table 7 in the Appendix. Interpretation of this table is similar to the other tables in the Appendix as described above.

### Estimation results

Results are presented in Table 3. Table 3 is subdivided into three panels (Movers & Stayers, Movers, Stayers) and results from all four matching estimators are reported in each panel. Each panel also provides baseline estimates from fixed and random effects regressions on the full sample of all control and treatment groups against which the DiD estimates can be compared. The DiD estimates for the unmatched samples (before we perform matching) are also reported in Table 3 is the coefficient estimate for the interaction term *b*_3_ in estimate for the interaction term *b*_3_ in Eq. 1. Standard errors in the table are robust standard errors. Bootstrapped standard errors, as suggested by Bertrand et al. [32], were calculated as well but these only differ at the third decimal place.

**Table 3:** Panel, DiD and MDiD estimated effects

		Movers & Stayers				Movers only				Stayers only			
		Effect	SE	*p*-value	*N*	Effect	SE	*p*-value	*N*	Effect	SE	*p*-value	*N*
	Panel FE	−0.009	0.014	0.523	99644	−0.010	0.017	0.547	13888	−0.014	0.026	0.593	85756
	Panel RE	0.012	0.010	0.246	99644	−0.007	0.016	0.673	13888	0.028**	0.014	0.043	85756
NM	Non-Exp, FE	0.005	0.020	0.783	70204	0.017	0.025	0.483	10148	0.061	0.040	0.120	60056
	Non-Exp, RE	0.011	0.019	0.581	70204	0.018	0.025	0.476	10148	0.061	0.040	0.123	60056
	Exp, FE	0.004	0.022	0.845	29440	−0.013	0.035	0.716	3740	0.046	0.035	0.188	25700
	Exp, RE	−0.003	0.021	0.895	29440	−0.018	0.034	0.592	3740	0.049	0.035	0.165	25700
2NN-nc	Non-Exp, FE	0.055**	0.025	0.025	7452	0.013	0.030	0.669	4612	0.061	0.047	0.198	1916
	Non-Exp, RE	0.053**	0.024	0.029	7452	0.014	0.030	0.632	4612	0.060	0.048	0.208	1916
	Exp, FE	0.007	0.026	0.775	7148	−0.052	0.038	0.171	3088	0.017	0.044	0.707	2264
	Exp, RE	0.002	0.026	0.927	7148	−0.055	0.038	0.147	3088	0.018	0.044	0.687	2264
5NN-nc	Non-Exp, FE	0.038**	0.022	0.087	13368	0.024	0.027	0.373	6820	0.050	0.043	0.244	3352
	Non-Exp, RE	0.040**	0.022	0.067	13368	0.024	0.027	0.373	6820	0.051	0.043	0.241	3352
	Exp, FE	0.004	0.023	0.879	11736	−0.026	0.035	0.464	3524	0.046	0.039	0.240	3832
	Exp, RE	−0.001	0.023	0.956	11736	−0.031	0.035	0.376	3524	0.050	0.039	0.204	3832
2NN-1/4c	Non-Exp, FE	0.052**	0.025	0.036	7428	0.008	0.030	0.798	4584	0.054	0.051	0.291	1788
	Non-Exp, RE	0.050**	0.024	0.041	7428	0.009	0.030	0.752	4584	0.052	0.051	0.306	1788
	Exp, FE	0.005	0.026	0.844	7088	−0.051	0.038	0.183	3056	0.011	0.045	0.800	2188
	Exp, RE	0.000	0.026	0.991	7088	−0.054	0.038	0.153	3056	0.012	0.045	0.794	2188
5NN-1/4c	Non-Exp, FE	0.034	0.022	0.126	13336	0.020	0.027	0.460	6796	0.044	0.047	0.350	3184
	Non-Exp, RE	0.036	0.022	0.097	13336	0.020	0.026	0.458	6796	0.044	0.047	0.350	3184
	Exp, FE	0.001	0.024	0.977	11672	−0.025	0.036	0.487	3492	0.036	0.040	0.368	3744
	Exp, RE	−0.004	0.023	0.849	11672	−0.031	0.035	0.384	3492	0.039	0.040	0.326	3744

Notes: *NM* = no matching, *2NN-nc* = 2 Nearest Neighbors-no caliper, *5NN-nc* = 5 Nearest Neighbors-no caliper, *2NN-1/4c* = 2 Nearest Neighbors-caliper equal to 1/4 of the SD of the estimated propensity score, *5NN-1/4c* = 5 Nearest Neighbors-caliper equal to 1/4 of the SD of the estimated propensity score

Standard errors are robust standard errors

*(**) [***] Statistically significant at the 10 % (5 %) [1 %] level

The first obvious result is that dollar stores have a positive effect on BMI. This effect is statistically significant, however, only for the full ‘Movers & Stayers’ sample. The DiD estimates from the two nearest neighbor matching show that in terms of magnitude the effect is about 5/100 of a standard deviation. We do not observe a statistically significant effect when we split the sample between ‘Movers’ and ‘Stayers’. However, if one observes closely the magnitude of the DiD estimates for these subsamples, it is obvious that the DiD estimate for the full sample is almost entirely driven by the ‘Stayers’ group. This is because for the ‘Movers’ subsample we get an estimate close to zero, while for the ‘Stayers’ subsample the DiD estimates are close to 6/100 of a standard deviation.

The positive effect for the non-exposed treatment implies that when the child moves from a food environment with a dollar store to a food environment without a dollar store, BMI increases on average by 5/100 of a standard deviation. Given that, as discussed above, the effect seems to be totally driven by ‘Stayers’, this effect could as well be due to a dollar store shutting down in the proximity of a child’s residence.

Both our matching and DiD models include variables of economic development (e.g., number of convenience and grocery stores, proportion of the population with income below poverty etc.) to account for the effect of broad changes that occur with economic development in an attempt to disentagle their effects from the pure effect of dollar stores. However, given that these potential confounders are likely endogenous, one may worry about spillover bias.^8^ To rule out an effect of neighborhood deterioration we also estimate effects from models that omit the economic development variables. Results are shown in Table 4. Consistent with our previous results, we find a positive and statistically significant effect for the ‘Stayers’ subsample in the non-exposed treatment. Thus, endogenous economic development is likely not a factor adversely affecting our results and we can be more confident that the positive effect on BMI of a dollar store shutting down in a neighborhood is a clean effect.

**Table 4:** Panel, DiD and MDiD estimated effects (without economic development variables)

		Movers & Stayers				Movers only				Stayers only			
		Effect	SE	*p*-value	*N*	Effect	SE	*p*-value	*N*	Effect	SE	*p*-value	*N*
	Panel FE	−0.009	0.014	0.530	99644	−0.010	0.017	0.533	13888	−0.014	0.026	0.597	85756
	Panel RE	0.019*	0.010	0.069	99644	−0.005	0.016	0.739	13888	0.040***	0.014	0.004	85756
NM	Non-Exp, FE	0.006	0.020	0.769	70204	0.018	0.024	0.454	10148	0.061	0.041	0.133	60056
	Non-Exp, RE	0.007	0.020	0.708	70204	0.017	0.024	0.482	10148	0.061	0.041	0.137	60056
	Exp, FE	0.005	0.023	0.834	29440	−0.015	0.033	0.659	3740	0.047	0.035	0.181	25700
	Exp, RE	−0.003	0.022	0.872	29440	−0.019	0.033	0.560	3740	0.050	0.035	0.156	25700
2NN-nc	Non-Exp, FE	0.023	0.025	0.355	7516	0.045	0.030	0.134	4768	0.094**	0.046	0.043	2160
	Non-Exp, RE	0.022	0.025	0.375	7516	0.045	0.030	0.132	4768	0.092**	0.046	0.046	2160
	Exp, FE	0.017	0.024	0.491	7404	−0.014	0.035	0.679	3316	0.020	0.041	0.632	2492
	Exp, RE	0.015	0.024	0.534	7404	−0.017	0.035	0.617	3316	0.021	0.041	0.616	2492
5NN-nc	Non-Exp, FE	0.015	0.022	0.481	14084	0.030	0.026	0.240	7336	0.077*	0.044	0.077	4128
	Non-Exp, RE	0.016	0.022	0.465	14084	0.028	0.026	0.276	7336	0.076*	0.044	0.080	4128
	Exp, FE	0.003	0.024	0.907	12596	−0.015	0.035	0.662	3680	0.022	0.039	0.569	4628
	Exp, RE	−0.001	0.023	0.965	12596	−0.018	0.035	0.595	3680	0.024	0.039	0.537	4628
2NN-1/4c	Non-Exp, FE	0.023	0.025	0.359	7512	0.043	0.029	0.135	4748	0.094**	0.048	0.049	2148
	Non-Exp, RE	0.022	0.025	0.376	7512	0.044	0.029	0.132	4748	0.093*	0.048	0.052	2148
	Exp, FE	0.016	0.026	0.543	7392	−0.014	0.035	0.679	3316	0.020	0.041	0.632	2492
	Exp, RE	0.014	0.026	0.588	7392	−0.017	0.035	0.617	3316	0.021	0.041	0.616	2492
5NN-1/4c	Non-Exp, FE	0.016	0.023	0.476	14068	0.030	0.027	0.263	7296	0.078*	0.043	0.071	4092
	Non-Exp, RE	0.017	0.023	0.463	14068	0.028	0.027	0.298	7296	0.077*	0.043	0.074	4092
	Exp, FE	0.002	0.023	0.938	12584	−0.015	0.035	0.662	3680	0.020	0.038	0.593	4604
	Exp, RE	−0.002	0.023	0.926	12584	−0.018	0.035	0.595	3680	0.022	0.038	0.559	4604

Notes: *NM* = no matching, *2NN-nc* = 2 Nearest Neighbors-no caliper, *5NN-nc* = 5 Nearest Neighbors-no caliper, *2NN-1/4c* = 2 Nearest Neighbors-caliper equal to 1/4 of the SD of the estimated propensity score, *5NN-1/4c* = 5 Nearest Neighbors-caliper equal to 1/4 of the SD of the estimated propensity score

Standard errors are robust standard errors

*(**) [***] Statistically significant at the 10 % (5 %) [1 %] level

## Discussion and Conclusions

The growth of dollar stores is a matter of interest to those seeking to address unacceptably high rates of childhood obesity. These stores tend to target smaller communities and lower income areas within urban population centers, areas where children would otherwise be at greater risk for obesity. No other known study, however, has examined the effect of dollar stores on childhood obesity. Our main goal in this paper is to determine whether access to dollar stores is a significant driver of childhood obesity. This is an interesting and important research topic since there is a perception that dollar stores typically do not offer healthier food alternatives compared to the traditional supermarkets. In this study, we are able to measure access to dollar stores around children’s actual residences and control for other attributes of the food environment (i.e., other types of food stores). Our focus on the state of Arkansas is also noteworthy since it has one of the highest childhood obesity rates in the US. Additionally, Arkansas was the first state to legislatively mandate the measurement and collection of BMI for every public school student starting in 2004 and so these data provide a unique opportunity to study child weight status and potential factors that impact BMI.

Using a unique panel data and difference in differences estimation with unmatched and matched children, we find no evidence that the presence of dollar stores within a reasonably close proximity to the child’s residence can increase body mass index. In fact, we see an increase in BMI z-score when dollar stores leave a child’s neighborhood. However, this finding is based on a small number of individuals for whom a dollar store exited their neighborhood, a rare phenomenon in the period we study. One should also keep in mind that our results concern very specific age cohorts. In addition, we restricted our analysis to children in early elementary grades because the diets of these children are more likely to still be dictated by the adults in their lives. In older children, several other competing factors may be at play which could confound any attempt to identify the separate effect of dollar stores on health and diet.

While dollar stores lack the breadth of healthy food options typically found in supermarkets, our results suggest that they are not a contributor to the childhood obesity problem. As noted above, the emergence of dollar stores as a common retail format is a recent phenomenon. It could be that these stores and their food inventories reflect existing preferences of the populations they serve. Thus, although dollar stores are more prominent in states like Arkansas, with high rates of obesity, they could be a symptomatic as opposed to a causal factor. Our results are consistent with this argument. Alternatively, it could be that dollar stores may actually play some role in facilitating healthy food consumption behaviors. Stambuck [11] inventoried several Arkansas dollar stores. The inventory revealed a dearth of fresh foods, especially fruits and vegetables, and very few low-sodium or reduced fat options. However these stores did provide healthy staple items such as dried beans, rice, and oatmeal. Many of the food items in dollar stores are packaged in a manner for at-home consumption. Hence, when residents have ready access to dollar stores, they may be in a better position to procure supplies for at-home meals. These meals, even if not perfectly balanced, are likely to be healthier and lower-calorie than the fare found on fast-food value menus.

Community leaders and public health professionals interested in childhood obesity would be wise to recognize that dollar stores are now prominent features of the food environment facing residents in many rural and lower income urban communities. As discussed earlier, many people now consider dollar stores as their neighborhood supermarkets. Dollar stores are especially dense in regions of the country where childhood obesity rates are the highest. The question of how dollar stores could contribute to dietary health should be considered in efforts to combat childhood obesity. For instance, educational interventions targeting children and their parents could emphasize ways to shop wisely at dollar stores to source nutritious food items. Community initiatives could also be developed that could further entice dollar stores to carry healthy foods. This would likely require cooperation between the store owners and the entire community. Moreover, as dollar stores continue to expand their food offerings, health on a budget may be a yet-to-be exploited marketing angle for this growing retail format.

## Endnotes

^1^ A quick overview of food access measures and definitions can be found at http://www.ers.usda.gov/data-products/food-access-research-atlas/about-the-atlas.aspx.

^2^ Recall that the periodicity of assessments was changed from all grades to even grades only, beginning in 2007 so that each age cohort was observed four times (with gaps) during this five-year period.

^3^ These include the proportion of block group residents that are African-American, Hispanic/Latino, that have completed high school, some college, or have attained a college degree. Block group measures also include proportion of the population with income below poverty, the median household income, the median age of residential housing stock, and the proportion of residential units that are vacant. We also include the proportion of single-parent families, working mothers, residents with no vehicles, and of residents using public transportation. Millimet and Tchernis [33] showed that over-specifying the model used to estimate the propensity score is always the best strategy, considering the penalty associated with under-specification. The rationale for including a control for language spoken at home, in addition to controls for race and ethnicity, is that recent immigrant families often have low socioeconomic status and may have different dietary behaviors than the population at large.

^4^ To make this statement clear, the model where the dependent variable is whether a dollar store is within a given radius (ten miles for rural areas and one mile for urban areas) from a child’s residence includes four additional covariates: (a) the log of the ratio of distance to a convenience store over distance to a dollar store (b) the log of the ratio of distance to a grocery store over distance to a dollar store (c) number of convenience stores within a ten (one) mile radius when the child resides in a rural (urban) area (d) number of grocery stores within a ten (one) mile radius when the child resides in a rural (urban) area.

^5^ The caliper width of 1/4, has been widely suggested in the PSM literature since Rosenbaum and Rubin [34]. Rosenbaum and Rubin [34] based this rule on results from Cochran and Rubin [35] that indicated that a caliper width of 1/4 of the standard deviation of the estimated propensity score would remove at least 90 % of the bias in a normally distributed covariate.

^6^ Mean standardized percent absolute bias is the mean absolute bias of the percent difference of the sample means in the treated and non-treated sub-samples as a percentage of the square root of the average of the sample variances in the treated and non-treated groups [34]. The percent bias is first calculated for each covariate separately and then the absolute values are averaged across all covariates and reported in Table 5 in the Appendix.

^7^ There is a 1:1 correspondence between Table 5 and Table 6 in the Appendix. To illustrate this, consider the non-exposed treatment that was matched with the 2 nearest neighbor (without caliper) matching estimator. Table 6 in the Appendix indicates that 423, 963 and 477 (Total = 1863) observations were retained after matching for the age cohorts 2004, 2005 and 2006, respectively. The number of retained observations corresponds to the sum of treated and control units (660 + 1203) in the respective rows and columns of Table 5 in the Appendix.

^8^ It has been shown that high-poverty neighborhoods have lower retail employment density for retail overall as well as several other types of retail, such as supermarkets, drugstores, food service and laundry [36]. On the other hand, neighborhoods that experience income upgrading see larger gains in retail employment.

## Appendix

**Table 5:** Balancing tests

			Movers & Stayers				Movers				Stayers			
			*p*-value	Mean % bias	*N* treated	*N* control	*p*-value	Mean % bias	*N* treated	*N* control	*p*-value	Mean % bias	*N* treated	*N* control
2 NN-nc	Non-Exp	BM	<0.001	8.37	660	16891	0.034	5.08	473	2064	<0.001	17.65	187	14827
		AM	0.999	2.51	660	1203	0.995	3.58	472	681	0.978	4.77	186	292
	Exp	BM	<0.001	13.19	684	6676	<0.001	10.80	463	469	<0.001	17.37	221	6204
		AM	0.975	2.83	684	1103	0.582	6.58	463	309	0.830	7.50	221	345
5NN-nc	Non-Exp	BM	<0.001	8.37	660	16891	0.034	5.08	473	2064	<0.001	17.65	187	14827
		AM	1.000	2.24	660	2682	0.978	3.24	472	1233	0.860	6.93	187	651
	Exp	BM	<0.001	13.19	684	6676	<0.001	10.80	463	469	<0.001	17.37	221	6204
		AM	0.243	5.13	684	2250	0.001	8.77	463	418	0.799	7.38	221	737
2 NN-1/4c	Non-Exp	BM	<0.001	8.37	660	16891	0.034	5.08	473	2064	<0.001	17.65	187	14827
		AM	0.996	2.63	657	1200	0.997	3.62	468	678	0.997	4.34	161	285
	Exp	BM	<0.001	13.19	684	6676	<0.001	10.80	463	469	<0.001	17.37	221	6204
		AM	0.993	2.49	673	1099	0.709	6.25	455	309	0.992	5.85	209	338
5NN-1/4c	Non-Exp	BM	<0.001	8.37	660	16891	0.034	5.08	473	2064	<0.001	17.65	187	14827
		AM	1.000	2.29	657	2677	0.993	3.10	468	1231	0.993	4.73	162	634
	Exp	BM	<0.001	13.19	684	6676	<0.001	10.80	463	469	<0.001	17.37	221	6204
		AM	0.425	4.68	673	2245	0.004	8.44	455	418	0.982	5.55	209	727

Notes: *Non-exp* = non exposed, *Exp* exposed, *BM* = Before matching, *AM* = After matching, *2NN-nc* = 2 Nearest Neighbors-no caliper, *5NN-nc* = 5 Nearest Neighbors-no caliper, *2NN-1/4c* = 2 Nearest Neighbors-caliper equal to 1/4 of the SD of the estimated propensity score, *5NN-1/4c* = 5 Nearest Neighbors-caliper equal to 1/4 of the SD of the estimated propensity score, *Mean %* |*b* *i* *a* *s*|= mean standardized % absolute bias, *N treat* = N of observations in the treated group, *N ctrl* = N of observations in the control group

*Mean standardized % absolute bias* is the mean absolute bias of the % difference of the sample means in the treated and non-treated sub-samples as a percentage of the square root of the average of the sample variances in the treated and non-treated groups [34]

*p*-values are the *p*-values from a likelihood-ratio test of the joint significance of all the regressors (before and after matching)

**Table 6:** Number of observations Dropped and Retained per cohort and matching estimator

			2004 cohort		2005 cohort		2006 cohort	
			Dropped	Retained	Dropped	Retained	Dropped	Retained
Movers & Stayers	2NN-nc	Non-exp	5114	423	5031	963	5543	477
		Exp	1675	620	1853	752	2045	415
	5NN-nc	Non-exp	4728	809	4347	1647	5134	886
		Exp	1318	977	1372	1233	1736	724
	2NN-1/4c	Non-exp	5114	423	5033	961	5547	473
		Exp	1677	618	1859	746	2052	408
	5NN-1/4c	Non-exp	4729	808	4349	1645	5139	881
		Exp	1320	975	1378	1227	1744	716
Movers	2NN-nc	Non-exp	442	337	485	483	457	333
		Exp	51	259	72	290	40	223
	5NN-nc	Non-exp	281	498	262	706	289	501
		Exp	11	299	24	338	19	244
	2NN-1/4c	Non-exp	444	335	488	480	459	331
		Exp	51	259	74	288	46	217
	5NN-1/4c	Non-exp	283	496	264	704	291	499
		Exp	11	299	26	336	25	238
Stayers	2NN-nc	Non-exp	4737	21	4643	383	5155	75
		Exp	1772	213	1935	308	2152	45
	5NN-nc	Non-exp	4726	32	4367	659	5083	147
		Exp	1638	347	1707	536	2122	75
	2NN-1/4c	Non-exp	4750	8	4657	369	5160	70
		Exp	1176	209	1937	306	2165	32
	5NN-1/4c	Non-exp	4744	14	4381	645	5093	137
		Exp	1642	343	1709	534	2138	59

Notes: *Non-exp* = non exposed, *Exp* = exposed, *2NN-nc* = 2 Nearest Neighbors-no caliper, *5NN-nc* = 5 Nearest Neighbors-no caliper, *2NN-1/4c* = 2 Nearest Neighbors-caliper equal to 1/4 of the SD of the estimated propensity score, *5NN-1/4c* = 5 Nearest Neighbors-caliper equal to 1/4 of the SD of the estimated propensity score

**Table 7:** Balancing tests for the unmatched data

		Fixed effects		Random effects		*N*	
		*p*-value	Mean % |*b* *i* *a* *s*|	*p*-value	Mean % |*b* *i* *a* *s*|	Treated	Control
Movers & Stayers	Non-exp	<0.001	12.31	<0.001	10.85	2640	67564
	Exp	<0.001	12.90	<0.001	11.68	2736	26704
Movers	Non-exp	<0.001	17.33	<0.001	13.65	1892	8256
	Exp	<0.001	16.03	<0.001	13.85	1852	1888
Stayers	Non-exp	<0.001	6.46	<0.001	7.97	748	59308
	Exp	<0.001	9.15	<0.001	10.21	884	24816

Notes: *Non-exp* = non exposed, *Exp* = exposed, *Mean % |bias|* mean standardized % absolute bias, *Treated* = N in the treated group, *Control* = N in the control group

*Mean standardized bias* is the % difference of the sample means in the treated and non-treated sub-samples as a percentage of the square root of the average of the sample variances in the treated and non-treated groups [34]

*p*-values are the *p*-values from a likelihood-ratio test of the joint significance of all the regressors

**Table 8:** Descriptive statistics for the Exposed and Non-exposed treatments

	Exposed			Non exposed		
	Mean treated	Mean control	*p*-value	Mean treated	Mean control	*p*-value
Low income	0.370	0.271	<0.001	0.367	0.372	0.74
Female	0.528	0.481	<0.001	0.542	0.491	<0.001
Age (in months)	91.522	91.604	0.89	91.503	91.469	0.95
Urban	0.787	0.832	<0.001	0.723	0.503	<0.001
Black/African-American	0.252	0.190	<0.001	0.264	0.218	<0.001
Hispanic/Latino	0.059	0.056	0.68	0.077	0.089	0.18
Native	0.003	0.004	0.43	0.003	0.004	0.56
Spanish language	0.048	0.045	0.59	0.063	0.074	0.19
Free lunch	0.454	0.299	<0.001	0.495	0.433	<0.001
Reduced lunch	0.106	0.091	0.09	0.089	0.103	0.14
% no vehicle	0.071	0.054	<0.001	0.067	0.070	0.25
% public transport	0.006	0.004	0.01	0.007	0.005	<0.001
% high-school	0.338	0.320	<0.001	0.354	0.370	<0.001
% some college	0.274	0.276	0.46	0.271	0.270	0.65
% more than college	0.201	0.248	<0.001	0.178	0.157	<0.001
% Hispanic/Latino	0.059	0.047	<0.001	0.068	0.064	0.27
% Black/African-American	0.202	0.160	<0.001	0.180	0.175	0.53
% single-parent families	0.288	0.234	<0.001	0.276	0.270	0.40
% income below poverty	0.182	0.151	<0.001	0.183	0.186	0.50
Median income (in thousands of $)	40.839	48.642	<0.001	40.920	40.133	0.09
% working mother	0.272	0.225	<0.001	0.268	0.256	0.08
Median home value (in thousands of $)	104.57	127.67	<0.001	100.56	97.03	0.01
Median age of residential housing stock	1979.20	1981.10	<0.001	1978.80	1978.80	0.98
% vacant residential units	0.116	0.114	0.56	0.132	0.122	<0.001

Notes: *p*-value is the *p*-value from a t-test of equality of means between treated and control

## References

[1] Ogden CL, Carroll MD, Curtin LR, Lamb MM, Flegal KM (2010). Prevalence of high body mass index in US children and adolescents, 2007–2008. JAMA.

[2] Ogden CL, Flegal KM, Carroll MD, Johnson CL (2002). Prevalence and trends in overweight among US children and adolescents, 1999–2000. JAMA.

[3] Arkansas Center for Health Improvement (ACHI) (2012). Year nine assessment of childhood and adolescent obesity in arkansas (fall 2011 - spring 2012).

[4] Institute of Medicine (IOM) (2012). Accelerating progress in obesity prevention: Solving the weight of the nation.

[5] Frieden TR, Dietz W, Collins J (2010). Reducing childhood obesity through policy change: Acting now to prevent obesity. Health Affairs.

[6] Goldberg ME, Gunasti K (2007). Creating an environment in which youths are encouraged to eat a healthier diet. J Public Policy & Mark.

[7] Story M, Kaphingst KM, Robinson-O’Brien R, Glanz K (2008). Creating healthy food and eating environments: Policy and environmental approaches. Ann Rev Public Health.

[8] Dunn RA (2010). The effect of fast-food availability on obesity: An analysis by gender, race, and residential location. Am J Agric Econ.

[9] Fan M, Jin Y (2013). Do neighborhood parks and playgrounds reduce childhood obesity?. Am J Agric Econ.

[10] Courtemanche C, Carden A (2011). Supersizing supercenters? The impact of walmart supercenters on body mass index and obesity. J Urban Econ.

[11] Stambuck H. The american diet: Surviving a meat-sweet desert: University of Arkansas Research Frontiers; 2013. Available at http://researchfrontiers.uark.edu/2012/the-american-diet-surviving-a-meat-sweet-desert/, (last accessed November 27, 2015).

[12] Florida R. What dollar store locations reveal about america. 2012. Available at http://www.citylab.com/work/2012/02/what-dollar-store-locations-reveal-about-america/1115/. (last accessed February 19, 2015).

[13] Natunewicz AT. Dollar days: How dollar stores are growing in a weak economy: Colliers International White Paper; 2011. Available at http://www.colliers.com/en-us/~/media/files/global/researchreports/colliers_whitepaper_dollardays_120611.ashx, (last accessed November 27, 2015).

[14] Benshimol Severin C, Benson-Armer R, Martinez A, Moore P. Entrenched frugality? The American consumer today: Technical report, McKinsey & Company; 2011. Available at http://www.mckinsey.com/~/media/mckinsey/dotcom/client_service/consumer%2520packaged%2520goods/pdfs/783827_entrenched_frugality_the_american_consumer_today1.ashx (last accessed November 27, 2015).

[15] IBISWorld Inc.Dollar & variety stores market research report: Technical report, IBISWorld Inc. Industry Report 45299; 2014. https://www.ibisworld.com/industry/default.aspx?indid=1093, (last accessed November 27, 2015).

[16] NPD Group. Millennial shoppers increase overall brick-and-mortar visits. 2014. Available at https://www.npd.com/wps/portal/npd/us/news/press-releases/dollar-stores-see-largest-increase-in-shopping-visits-among-brick-and-mortar-retailers/, (last accessed November 27, 2015).

[17] National Research Council (2010). Bridging the evidence gap in obesity prevention: A framework to inform decision making.

[18] Justus MB, Ryan KW, Rockenbach J, Katterapalli C, Card-Higginson P (2007). Lessons learned while implementing a legislated school policy: Body mass index assessments among Arkansass public school students. J School Health.

[19] Khandker SR, Koolwal GB, Samad HA (2010). Handbook on Impact Evaluation: Quantitative Methods and Practices.

[20] Angelucci M, Attanasio O (2013). The demand for food of poor urban mexican households: Understanding policy impacts using structural models. Am Econ J Econ Policy.

[21] Racine EF, Lyerly J, Troyer JL, Warren-Findlow J, McAuley WJ (2012). The influence of home-delivered dietary approaches to stop hypertension meals on body mass index, energy intake, and percent of energy needs consumed among older adults with hypertension and/or hyperlipidemia. J Acad Nutr Diet.

[22] Besley T, Case A (2000). Unnatural experiments? Estimating the incidence of endogenous policies. Econ J.

[23] Abadie A (2005). Semiparametric difference-in-differences estimators. Rev Econ Stud.

[24] Blundell R, Duncan A, Meghir C (1998). Estimating labor supply responses using tax reforms. Econometrica.

[25] Campbell BL, Nayga RM, Park JL, Silva A (2011). Does the national school lunch program improve childrens dietary outcomes?. Am J Agric Econ.

[26] Islam A (2011). Medium- and long-term participation in microcredit: An evaluation using a new panel dataset from bangladesh. Am J Agric Econ.

[27] Heckman JJ, Ichimura H, Todd PE (1997). Matching as an econometric evaluation estimator: Evidence from evaluating a job training programme. Rev Econ Studies.

[28] Blundell R, Dias MC (2000). Evaluation methods for non-experimental data. Fiscal Studies.

[29] Blundell R, Dias MC, Meghir C, van Reenen J (2004). Evaluating the employment impact of a mandatory job search program. J Eur Econ Assoc.

[30] Lechner M (2008). A note on endogenous control variables in causal studies. Stat Probab Lett.

[31] Kuczmarski RJ, Ogden CL, Guo SS, Grummer-Strawn LM, Flegal KM, Mei Z, et al. 2000 CDC growth charts for the united states: Methods and development. Vital Health Stat. 2002;11(246). 12043359

[32] Bertrand M, Duflo E, Mullainathan S (2004). How much should we trust differences-in-differences estimates?. Q J Econ.

[33] Millimet DL, Tchernis R (2009). On the specification of propensity scores, with applications to the analysis of trade policies. J Business Econ Stat.

[34] Rosenbaum PR, Rubin DB (1985). Constructing a control group using multivariate matched sampling methods that incorporate the propensity score. Am Stat.

[35] Cochran WG, Rubin DB (1973). Controlling bias in observational studies: A review. Sankhyā: Indian J Stat Series A.

[36] Schuetz J, Kolko J, Meltzer R (2012). Are poor neighborhoods “retail deserts”?. Reg Sci Urban Econ.

